# Golfers’ Interest in Multilevel Sun-Protection Strategies

**DOI:** 10.3390/ijerph18147253

**Published:** 2021-07-07

**Authors:** Amanda E. Weikert, Sherry L. Pagoto, Eric Handley, Jimikaye B. Courtney, Deborah Brunke-Reese, David E. Conroy

**Affiliations:** 1 Department of Kinesiology, The Pennsylvania State University, University Park, PA 16802, USA; aew5459@psu.edu (A.E.W.); jimikaye@psu.edu (J.B.C.); dlb43@psu.edu (D.B.-R.); 2 Department of Allied Health Sciences, University of Connecticut, Storrs, CT 06269, USA; Sherry.Pagoto@uconn.edu; 3 Golf Teaching and Research Center, The Pennsylvania State University, University Park, PA 16082, USA; esh12@psu.edu; 4 Department of Preventive Medicine, Northwestern University, Chicago, IL 60611, USA

**Keywords:** sun protection, skin cancer prevention, physical activity, primary prevention

## Abstract

Active adults accumulate more ultraviolet (UV) radiation exposure and are at greater risk of skin cancer than inactive adults. Golf is a popular sport that increases UV exposure because it is played outdoors in daylight. This study evaluated adult golfers’ interest in multilevel sun-protection strategies and characterized differences in interest as a function of golfer characteristics. Adult golfers (*N* = 347) completed a web survey to rate their interest in 20 sun-protection strategies. We estimated descriptive statistics and evaluated differences in interest as a function of demographics, perceived risk, sun-protective behavior, and golf exposure. Golfers reported the greatest interest in environmental supports for sun protection, but these ratings were driven by golfers who already perceived golf as a risk behavior and used sunscreen diligently. Vulnerable golfers—those with a golf-related sunburn in the past year or who spend more time golfing—expressed interest in a broader range of intervention components, including education, family support, and text messages. Multilevel skin cancer prevention interventions are needed for golfers. Intervention components of interest involved support and reminders, which suggests they are open to sun-safety behaviors but need help executing them.

## 1. Introduction

Physical activity reduces risk for many types of cancer but has been linked to 27% greater risk for skin cancer, likely due to active adults accumulating more ultraviolet (UV) radiation exposure than inactive adults [1,2,3]. Exposure-related risks can be reduced by seeking shade and limiting time in the sun during peak hours of UV radiation, wearing protective clothing (e.g., sleeves, pants, hats) and sunglasses, and wearing sunscreen (sun-protection factor 15 or higher) [4]. Yet, sun-protective behavior tends to be poor during warm-weather leisure activities [5]. Interventions have proven valuable for promoting sun protection in some recreational contexts. For example, sun-protection interventions have been developed for children in aquatic environments and vacationers at waterfronts of warm-weather resorts [6,7]. We are not aware of any evidence-based sun-protection interventions to reduce sunburn and prevent skin cancer in golfers. As a first step toward intervention development, this study investigated adult golfers’ interest in intervention components for promoting sun protection while golfing.

Golf is a type of physical activity that requires particular attention for skin cancer prevention because it is popular and played outdoors during peak UV hours. In the United States, the National Golf Foundation estimated that over 24 million Americans (age 6 and older) played on a golf course in 2019 and that over 440 million rounds of golf were played that year [8]. To put those numbers in context, only California and Texas have state populations in the United States that exceed 24 million [9]. At 3–4 hours per round, Americans are accumulating in excess of 1.3 billion hours of UV exposure per year. Additionally, over 75% of golfers report having fair or very fair skin, which heightens their risk for sunburn, a biomarker of risk for melanoma, the deadliest form of skin cancer [10,11]. The top of the head receives greater exposure during golf than the upper back/shoulders or arms [12]. The intensity of UV exposures from golf appears to increase skin cancer risk at younger ages, as evidenced by comparisons in a small sample of younger professional vs. older amateur golfers [13].

Sun-protective behaviors can mitigate risk for both sunburn and skin cancer in golfers. The most common sun-protective behaviors reported by golfers include wearing a hat (69%), wearing sunscreen (66%), wearing sunglasses (56%), or seeking shade (45%) [10]. Golfers were less likely to avoid sun exposure during midday hours (30%) or to wear long-sleeved shirts or long pants (8%). Clothing is effective for reducing UV exposure, but those effects are specific to the parts of the body covered by the clothing [14]. Exposure may also vary by location on a golf course, with putting greens potentially presenting greater risk due to limited shade.

Interventions to promote sun protection in golfers could engage multiple levels of the social-ecological model [15]. For example, the built environment could be enhanced by making sunscreen readily available in the clubhouse and on the course and by promoting sun-safe apparel, sunglasses, and hats in pro shops. Information about the natural environment could be used to increase golfers’ awareness of daily UV index forecasts that would signal risk and elevated need for sun protection. The digital environment could be engaged by developing a website to educate golfers about sun protection and skin cancer risk, using text messages to prompt golfers to protect their skin when golfing (particularly on high-risk days), or supporting monitoring of UV exposure with sensors (e.g., connected devices, temporary tattoos). The interpersonal environment could be targeted by engaging golf pros and family members as champions or social supporters in a sun-safe golf campaign. Many of these intervention components are likely to be sustainable because they could create revenue lines via sales or sponsorships. Yet, it is unclear which components might interest golfers, and interest in innovations can enhance implementation success [16]. It is also unclear which components might appeal to the most vulnerable golfers who could benefit the most from a sun-protection intervention. Identifying these factors can inform selection of components for a skin cancer prevention intervention designed to appeal to golfers.

The purpose of this study was to characterize adult golfers’ interest in a variety of intervention components that could be used to promote sun safety. A second objective was to identify components that held the most appeal to golfers at higher risk for skin cancer. Indicators of golfers at higher risk included demographic characteristics (e.g., age, sex), perceived risk (e.g., skin type, past-year sunburn), using sun-protective behaviors (e.g., applying and re-applying sunscreen, wearing sleeves and hats, seeking shade), and golf-related exposure (e.g., lifetime and past-year golfing).

## 2. Materials and Methods

Adult golfers in the United States were recruited via a Qualtrics panel between 27 May and 3 June 2020 (*N* = 366). Eligibility criteria included 18–70 years of age and having played at least 18 holes of golf per month in the previous summer. Sampling quotas were prespecified to achieve a rectangular age distribution from 18–70 years and to represent the U.S. adult population in terms of sex, race, and ethnicity. This dataset has been used for one other study on alcohol use in golfers, but all of the data and analyses reported in this study are novel [17].

Eligibility was screened using two questions: “Are you between the ages of 18 and 70 years?” and “Last summer, on average, did you play at least 18 holes/month in June, July, and August 2019?” Participants were eligible if both responses were affirmative.

Demographic characteristics assessed in the survey included age, sex, race, ethnicity, marital status, employment status, and highest level of education. Skin type was assessed using the Fitzpatrick scale [18]. Participants reported on the frequency of past-year experiences with a red or painful sunburn that lasted a day or more [19]. Perceived skin cancer risk was assessed with a single item, “To what extent do you feel at risk for skin cancer?” (rated from 0 (no risk) to 10 (high risk)) [2]. Perceptions of golf as a risk factor were assessed with a single item, “To what extent do you feel playing golf alters your risk for skin cancer?” (rated from 1 (reduces risk a lot) to 5 (increases risk a lot)).

Sun-protective behaviors (wearing sunscreen, wearing a shirt with sleeves that cover the shoulders, wearing a hat, and staying in the shade or under an umbrella) during golf outside during the summer on a warm sunny day were assessed using five items from the Sun-Protection Habits scale (ratings from 0 (never) to 5 (always)) [19]. Golf experience was assessed as the number of years participants had been golfing (response options: <1 year, 1–5 years, 6–10 years, 11–15 years, and 15+ years). Participants also rated their golf activity in the past three seasons excluding winter, with questions about frequency (days/week), volume (holes/week), and duration of practice time on putting green or driving range (hours/week). Seasons were defined as spring (March–May 2019), summer (June–August 2019), and fall (September–November 2019).

Twenty potential intervention components for promoting sun protection in golfers were listed. Participants rated their interest in each item on a four-point scale ranging from 0 (not at all interested) to 3 (extremely interested; intermediate options labeled slightly interested and moderately interested).

Prior to analysis, 14 participants were removed due to unusual response patterns (11 nonsensical responses to the write-in variable for occupation, three implausible health histories) or reporting playing no golf from March to November 2019 (*n* = 5). The analytic sample included 347 cases (95% of all cases). IBM SPSS Statistics for Windows, version 26 (IBM Corp., Armonk, NY, USA) was used to calculate descriptive statistics, Spearman’s rho (ρ) correlations (for continuous variables), and Kruskal–Wallis H tests (for categorical variables). The type-1 error level was adjusted to 0.0025 because each independent variable was tested with 20 intervention components.

## 3. Results

Sample characteristics are summarized in Table 1. Most participants believed that golf increased their risk for skin cancer a little or a lot (*n* = 211 (61%)), but some believed that golf either did not alter their risk (*n* = 86 (25%)) or reduced their risk by a little or a lot (*n* = 50 (14%)). Most of the sample (*n* = 244 (70%)) had one or more red or painful sunburns that lasted a day or more in the past year due to a combination of golf (*n* = 200 (58%)) and non-golf activities (*n* = 230 (66%)).

Table 2 summarizes the frequencies that golfers reported using common sun-protective behaviors. Many golfers always or often wore sunscreen (59%), sleeves that covered their shoulders (65%), or a hat while golfing (68%). Few golfers (33%) often or always stayed in the shade or under an umbrella while golfing. Golf experience was collapsed into three categories: ≤5 years (*n* = 90 (6%)), 6–15 years (*n* = 109 (31%)), or 15+ years (*n* = 148 (43%)). Across the spring, summer, and fall, participants reported playing golf (M ± SD) 1.9 ± 1.7 times/week for an average of 23.0 ± 13.1 holes/week and practicing on a driving range or putting green for 1.8 ± 1.7 h/week. Assuming that 9 holes of golf take approximately 2.25 h, these responses equate to over 7.5 h/week of potential UV exposure.

Table 3 summarizes golfers’ interest in each of the 20 intervention components (categories for not at all or slightly interested were collapsed). Golfers most frequently reported moderate or extreme interest in apparel (sunglasses and UV-protective shirts) and environmental supports (sunscreen dispensers and signs). All components attracted at least moderate or extreme interest from approximately half of the sample, and none were unequivocally rejected.

Table 4 summarizes ratings that varied significantly as a function of demographic characteristics, perceived risk, sun-protective behaviors, and golf-related exposures (in that order).

Older golfers were more interested in sunscreen dispensers on the course (ρ = 0.20, *p* = 0.0002). Younger golfers were more interested than older golfers in text messages (UV risk notification ρ = −0.25, *p* < 0.0001; sun protection reminders ρ = −0.23, *p* < 0.0001), umbrellas for their carts (ρ = −0.25, *p* < 0.0001), seeing examples of sun-damaged skin (ρ = −0.22, *p* < 0.0001), and social support for sun protection (family support ρ = −0.20, *p* = 0.0020; golf pro support ρ = −0.17, *p* < 0.0001). Men reported greater interest in family support than women, χ^2^(1) = 11.59, *p* < 0.0001.

Golfers whose skin type burned easily/always expressed greater interest in brochures (χ^2^(2) = 31.68, *p* < 0.0001), websites (χ^2^(2) = 17.29, *p* = 0.0002), and text messages about daily UV risk (χ^2^(2) = 13.69, *p* = 0.0011) than did golfers who burned less easily. Golfers who perceived a greater risk were more interested in wide-brim hats (ρ = 0.21, *p* < 0.0001), educational brochures (ρ = 0.18, *p* = 0.0008), and sunscreen dispensers in the clubhouse (ρ = 0.18, *p* = 0.0011). Golfers with stronger beliefs that golfing increased their risk reported greater interest in free or discounted UV-protective golf shirts (χ^2^(1) = 18.8, *p* < 0.0001), signs with UV index on the course (χ^2^(1) = 10.6, *p* = 0.0012), and sunscreen dispensers on the course (χ^2^(1) = 10.0, *p* = 0.0015) than golfers who did not believe that golf increased their risk.

Golfers who had one or more sunburns from golfing reported greater interest in ten components than golfers who did not have a sunburn from golfing in the past year: text message reminders to use sun protection (χ^2^(1) = 39.3, *p* < 0.0001), text messages indicating daily UV risk (χ^2^(1) = 29.8, *p* < 0.0001), seeing an example of how sun damage will age their appearance (χ^2^(1) = 18.0, *p* < 0.0001), reminders from family members (χ^2^(1) = 18.1, *p* < 0.0001), signs with sun-protection reminders in the clubhouse (χ^2^(1) = 13.5, *p* = 0.0002), signs with sun-protection reminders on the course (χ^2^(1) = 12.7, *p* = 0.0004), mounted umbrellas for carts (χ^2^(1) = 11.1, *p* = 0.0009), signs in the clubhouse with the UV forecast (χ^2^(1) = 11.0, *p* = 0.0009), an educational website (χ^2^(1) = 10.4, *p* = 0.0013), and an educational brochure (χ^2^(1) = 9.22, *p* = 0.0024). Golfers who had one or more sunburns from non-golf activities reported greater interest in five components than golfers who did not have a sunburn from non-golf activities: text message reminders to use sun protection (χ^2^(1) = 16.2, *p* < 0.0001), text messages indicating daily UV risk (χ^2^(1) = 14.6, *p* = 0.0001), seeing an example of how sun damage will age their appearance (χ^2^(1) = 15.8, *p* < 0.0001), reminders from family members (χ^2^(1) = 14.8, *p* = 0.0001), and mounted umbrellas for carts (χ^2^(1) = 9.2, *p* = 0.0024).

Golfers who always used sunscreen while golfing reported greater interest in sunscreen dispensers in the clubhouse (χ^2^(3) = 34.5, *p* < 0.0001), sunscreen dispensers on the course (χ^2^(3) = 33.7, *p* < 0.0001), signs with the UV forecast on the course (χ^2^(3) = 23.3, *p* < 0.0001), free or discounted UV-protective golf shirts (χ^2^(3) = 17.6, *p* = 0.0005), an educational website (χ^2^(3) = 15.0, *p* < 0.0018), and golf pros being role models (χ^2^(3) = 14.4, *p* = 0.0024) than were golfers who used sunscreen less frequently. Golfers who wore shoulder-covering sleeves more frequently were more interested in sunscreen dispensers on the course than those who wore shoulder-covering sleeves less frequently, χ^2^(3) = 29.9, *p* < 0.0001. Golfers who wore a hat sometimes or often were more interested in four components than golfers who work a hat either never/rarely or always: text messages indicating daily UV risk (χ^2^(3) = 20.1, *p* = 0.0002), text messages with sun-protection reminders (χ^2^(3) = 19.5, *p* = 0.0002), sunscreen dispensers in the clubhouse (χ^2^(3) = 16.3, *p* = 0.0010), and seeing an example of how sun damage will age their appearance (χ^2^(3) = 14.7, *p* = 0.0020). The frequency of staying in the shade or under an umbrella while golfing was not associated with golfers’ interest in any of the components.

Golfers with more than 15 years of experience reported less interest in umbrellas (χ^2^(2) = 17.99, *p* = 0.0001) and seeing examples of how sun-damage will age their appearance (χ^2^(2) = 13.95, *p* = 0.0009) than golfers with less experience. Golfers who played more days/week were more interested in five components: text messages (reminders about sun-protective behaviors: ρ = 0.21, *p* < 0.0001; daily UV risk: ρ = 0.16, *p* < 0.0023), reminders from family members (ρ = 0.17, *p* < 0.0011), seeing an example of how sun damage will age their appearance (ρ = 0.19, *p* = 0.0004), and educational brochures (ρ = 0.20, *p* = 0.0002). The duration of time practicing on the driving range or putting green each week was positively associated with interest in 10 components: seeing an example of how sun damage will age their appearance (ρ = 0.31, *p* < 0.0001), text message reminders about sun-protective behaviors (ρ = 0.27, *p* < 0.0001), umbrellas and mounts for push carts (ρ = 0.27, *p* < 0.0001), reminders from family members (ρ = 0.24, *p* < 0.0001), text messages indicating daily UV risk (ρ = 0.23, *p* < 0.0001), educational brochures (ρ = 0.23, *p* < 0.0001), UV-protective golf shirts (full price; ρ = 0.21, *p* = 0.0001), an educational website (ρ = 0.20, *p* = 0.0002), signs with sun-protection reminders in the clubhouse (ρ = 0.20, *p* = 0.0002), and disposable skin stickers or temporary tattoos that track UV exposure (ρ = 0.16, *p* = 0.0022). The number of holes played per week was not associated with interest in any of the components.

## 4. Discussion

Findings revealed that 39% of golfers did not believe that golfing increased their risk for skin cancer, which includes 14% who believed golfing lowers their risk for skin cancer. Golfers in general expressed broad interest in sun-protection intervention components that spanned multiple levels of the social-ecological model. Environmental supports for sun protection, both in the clubhouse and on the course, attracted considerable interest. Since 2015, the non-profit group IMPACT Melanoma has distributed free sunscreen dispensers in community settings, and sunscreen dispensers are appearing on some golf courses around the country [20,21,22]. Observational data from a large state fair indicated that public sunscreen dispensers were widely used, especially on sunny days and days with a high UV index [23]. The American Society for Dermatologic Surgery has promoted implementation of community sunscreen dispensers through their SPF for All program [24]. Sunscreen dispensers typically have signage space on them which presents opportunities to test different messaging strategies to increase use. Research is needed to evaluate the effects of dispensers on golf courses and, if effective, to develop strategies to increase the implementation of sunscreen dispensers on golf courses.

Golfers also specifically expressed interest in signage with the daily UV index forecast and reminders to use sun protection while playing. This strategy would align with prior work at warm-weather resorts and aquatic settings [6,7]. Prior work with office workers indicated that disseminating weekend UV forecasts via a late-week email did not increase reported sun protection [25]. This environmental information may be more useful on a just-in-time basis, such as in the clubhouse or on the course, when vulnerability will be high and action can be taken, as in prior studies in ski resort and vacation resort settings [6,26].

Environmental supports for sun protection held the greatest appeal to golfers who were already diligently using sunscreen when they played and believed that golf increased their risk for skin cancer. Two specific characteristics of golfers that increase risk can be leveraged to target a sun-protection intervention. Golfers with past-year sunburns from golfing and greater practice time expressed interest in intervention components that targeted a wide variety of social-ecological influences on sun protection. For example, high-risk golfers specifically expressed interest in print and electronic educational materials about sun safety for golfers, mirroring prior work in ski resort and vacation resort settings [6,26]. They also expressed interest in seeing examples of accelerated photoaging. The free Sunface mobile app simulates photoaging and has proven effective for increasing sunscreen use and decreasing tanning in high school students; it may also be useful for golfers [27,28]. Age may account for some of this interest because of heightened appearance motives and comfort with technology. The documented differences were modest in size, so although they warrant consideration, they should not be the sole factor used to select interventions to target high-risk golfers.

High-risk golfers also expressed greater interest in text message reminders about daily UV risk and sun-protective behaviors than did less vulnerable golfers. Existing trials of text message reminders for sun protection have a high risk of bias from randomization and outcome measurement methods, so conclusions should be drawn cautiously [29]. Messages may be more effective if they focus on mitigating risk on days with high UV forecasts or when people plan to play golf. If intervention efforts are targeted toward these high-risk golfers, they should include components to build motivation for sun-protective behaviors as well.

Almost all of these components can be implemented at golf clubs for minimal cost and, in some cases, a potential profit. A public zoo found that a multicomponent sun-protection intervention increased purchase rates for sunscreen and hats in the gift shop [30]. Sponsorships are also a possible avenue to offset costs. The IMPACT Melanoma program for sunscreen dispensers has increasingly been funded by public health departments, parks and recreation associations, and health care organizations [20]. Similar approaches should be explored to support golfers’ sun protection.

The sample was recruited to represent the age, sex, race, and ethnicity of the US population, but it is not necessarily a representative sample of golfers. The observational research design prevents us from inferring how golfer characteristics affect interest in intervention components. Self-reported interest also does not always correspond with uptake of intervention components or enactment of sun protection [31]. However, engaging golfers in the development of multi-level intervention strategies may increase the feasibility and acceptability of such strategies. Future work may benefit from a participatory action research approach that engages golfers in co-designing a sun-protection intervention.

## 5. Conclusions

In closing, warm-weather recreational activities have been a focus for skin cancer prevention efforts, but strategies have been more effective in waterside than dry-land settings [6,7,32,33]. Golfers in this study expressed a strong interest in apparel and environmental supports for sun protection, such as sunscreen dispensers and signage with sun-protection prompts or information about UV risk. High-risk golfers were interested in a broader range of intervention components that included education, family support, and text messages. These findings can inform the selection of components for multilevel sun-protection interventions. Given the millions of golfers who accumulate over one billion hours of UV exposure during golf each year and the lack of effective sun-protection programs for land-based, warm weather, recreational activities, a multilevel sun-protection intervention in this context would be valuable for skin cancer prevention. This study provides a foundation for selecting components at varying levels of influence.

## Figures and Tables

**Table 1:** Characteristics of golfers.

Characteristic	*n* (%)
**Sex**	
Female	171 (49.3)
Male	176 (50.7)
**Race**	
White	277 (79.8)
African-American or Black	33 (9.5)
Asian-American	22 (6.3)
American Indian/Alaska Native, Native Hawaiian/Other Pacific Islander, or two or more races	15 (4.3)
**Ethnicity**	
Not Hispanic or Latinx	310 (89.3)
Hispanic or Latinx	37 (10.7)
**Marital Status**	
Married/cohabitate	226 (65.1)
Never married	80 (23.1)
Widowed, divorced, or separated	41 (11.8)
**Employment Status**	
Employed, full-time	212 (61.1)
Retired	71 (20.5)
Employed, part-time	36 (10.4)
Not employed but looking, or never employed	28 (8.1)
**Highest Level of Education**	
Bachelor’s degree (e.g., BA, BS)	130 (37.5)
Some college, Associate’s degree, or no degree	101 (29.1)
Graduate or professional degree	82 (23.7)
No college education (GED, HS diploma, or dropout)	34 (9.8)
**Skin Type**	
Pale white skin, blue/green eyes, blond red hair (always burns, does not tan)	34 (9.8)
Fair skin, blue eyes (burns easily, tans poorly)	86 (24.8)
Darker white skin (tans after initial burn)	127 (36.6)
Light brown skin (burns minimally, tans easily)	71 (20.5)
Brown skin (rarely burns, tans darkly easily)	21 (6.1)
Dark brown or black skin (never burns, always tans darkly)	8 (2.3)

**Table 2:** Golfers’ use of sun-protective behaviors.

			*n* (%)		
Sun-Protective Behavior While Golfing	Never	Rarely	Sometimes	Often	Always
Wear sunscreen	20 (5.8)	43 (12.4)	78 (22.5)	81 (23.3)	125 (36)
Wear shirt with sleeves that cover shoulders	15 (4.3)	36 (10.4)	72 (20.7)	72 (20.7)	152 (43.8)
Wear a hat	21 (6.1)	26 (7.5)	65 (18.7)	77 (22.2)	158 (45.5)
Stay in the shade or under an umbrella	25 (7.2)	70 (20.2)	139 (40.1)	91 (26.2)	22 (6.3)

**Table 3:** Golfers’ interest in components of a sun-safety intervention.

Intervention Component	Not at All or Slightly Interested (*n* [%])	Moderately Interested (*n* [%])	Extremely Interested (*n* [%])
**Intraindividual components**			
Sunglasses providing UV protection	65 (18.7)	116 (33.4)	166 (47.8)
Sunscreen dispensers in the clubhouse	74 (21.3)	128 (36.9)	145 (41.8)
Free or discounted UV-protective golf shirts with sponsor logo	81 (23.3)	122 (35.2)	144 (41.5)
A device that clips to shirt collar or golf bag that senses the UV exposure you’ve had and can give you updates via a mobile app	147 (42.4)	123 (35.4)	77 (22.2)
Seeing an example of how sun damage will age my appearance	154 (44.4)	117 (33.7)	76 (21.9)
Website with information about sun safety for golfers	141 (40.6)	131 (37.8)	75 (21.6)
Wide brim hat	141 (40.6)	131 (37.8)	75 (21.6)
Disposable skin stickers or temporary tattoos or (free or low cost) that can reveal UV exposure by changing colors	151 (43.5)	124 (35.7)	72 (20.7)
Umbrella and mount for a push golf cart	155 (44.7)	122 (35.2)	70 (20.2)
Brochure with information about sun safety for golfers	160 (46.1)	123 (35.4)	64 (18.4)
UV-protective golf shirts (full price) without sponsor logo	158 (45.5)	126 (36.3)	63 (18.2)
**Interpersonal components**			
Golf pros being good role models for sun safety	132 (38.0)	124 (35.7)	91 (26.2)
My family helping me to remember to protect myself from the sun	138 (39.8)	140 (40.3)	69 (19.9)
**Built environment components**			
Sunscreen dispensers on the course (e.g., on 9th hole to remind you to reapply sunscreen after approximately 2 h of play)	101 (29.1)	114 (32.9)	132 (38.0)
Signs with sun-protection reminders in clubhouse	127 (36.6)	135 (38.9)	85 (24.5)
Signs with sun- protection reminders on the course	128 (36.9)	146 (42.1)	73 (21.0)
**Natural environment components**			
Signs with UV index forecast on the course	104 (30.0)	148 (42.7)	95 (27.4)
Signs in the clubhouse with UV forecast information	117 (33.7)	141 (40.6)	89 (25.6)
**Digital environment components**			
Text messages with information about daily UV risk	182 (52.4)	113 (32.6)	52 (15)
Text messages reminders on sun-protective behaviors	189 (54.5)	107 (30.8)	51 (14.7)

**Table 4:** Golfers’ interest in components of a sun-safety intervention.

Intervention Component	Greater Interest Among Those Who…
**Intraindividual components**	
Sunglasses providing UV protection	No differences
Free or discounted UV-protective golf shirts with sponsor logo	(1) view golf as increasing their risk for skin cancer, (2) use sunscreen more frequently
A device that clips to shirt collar or golf bag that senses the UV exposure you’ve had and can give you updates via a mobile app	No differences
Seeing an example of how sun damage will age my appearance	(1) are younger, (2) have less experience playing golf, (3) play golf more frequently, (4) spend more time practicing golf, (5) wear a hat more frequently, (6) have a history of more sunburns (golf- and non-golf related)
Website with information about sun safety for golfers	(1) have a fairer skin type, (2) have a history of more golf-related sunburns, (3) use sunscreen more frequently, (4) spend more time practicing golf
Wide-brim hat	(1) perceive themselves to be at risk for skin cancer
Disposable skin stickers or temporary tattoos or (free or low cost) that can reveal UV exposure by changing colors	(1) spend more time practicing golf
Umbrella and mount for a push golf cart	(1) are younger, (2) have less experience playing golf, (3) spend more time practicing golf, (4) have a history of more sunburns (golf- or non-golf related)
Brochure with information about sun safety for golfers	(1) have fairer skin, (2) perceive themselves to be at risk for skin cancer, (3) have a history of more golf-related sunburns, (4) play golf more frequently, (5) spend more time practicing golf
UV-protective golf shirts (full price) without sponsor logo	(1) spend more time practicing golf
**Interpersonal components**	
Golf pros being good role models for sun safety	(1) are younger, (2) use sunscreen more frequently
My family helping me to remember to protect myself from the sun	(1) are younger, (2) are male, (3) have a history of more golf-related sunburns, (4) play golf more frequently, (5) spend more time practicing golf
**Built environment components**	
Sunscreen dispensers in the clubhouse	(1) perceive themselves to be at risk for skin cancer, (2) use sunscreen more frequently, (3) wear a hat more frequently
Sunscreen dispensers on the course (e.g., on 9th hole to remind you to reapply sunscreen after approximately 2 h of play)	(1) are older, (2) believe golf increases risk for skin cancer, (3) use sunscreen more frequently, (4) wear long sleeves more frequently
Signs with sun-protection reminders in clubhouse	(1) have a history of more golf-related sunburns, (2) spend more time practicing golf
Signs with sun-protection reminders on the course	(1) have a history of more golf-related sunburns
**Natural environment components**	
Signs with UV index forecast on the course	(1) believe golf is a risk factor for skin cancer
Signs in the clubhouse with UV forecast information	(1) have a history of more golf-related sunburns, (2) use sunscreen more frequently
**Digital environment components**	
Text messages with information about daily UV risk	(1) are younger, (2) have fairer skin, (3) have a history of more sunburns (golf- and non-golf related), (4) wear a hat frequently, (5) play golf more frequently, (6) spend more time practicing golf
Text messages reminders on sun-protective behaviors	(1) are younger, (2) have a history of more sunburns (golf- and non-golf related), (3) wear a hat frequently, (4) play golf more frequently, (5) spend more time practicing golf

## Data Availability

Data are available from the corresponding author upon reasonable request.

## References

[1] Moore S.C., Lee I.-M., Weiderpass E., Campbell P.T., Sampson J.N., Kitahara C.M., Keadle S.K., Arem H., Berrington de Gonzalez A., Hartge P. (2016). Association of Leisure-Time Physical Activity with Risk of 26 Types of Cancer in 1.44 Million Adults. JAMA Intern. Med..

[2] Schneider K.L., Pagoto S., Panza E., Keeney J., Goldberg D. (2014). Skin Cancer Risk Profiles of Physically Active Adults. Health Behav. Policy Rev..

[3] Snyder A., Valdebran M., Terrero D., Amber K.T., Kelly K.M. (2020). Solar Ultraviolet Exposure in Individuals Who Perform Outdoor Sport Activities. Sports Med. Open.

[4] U.S. Department of Health and Human Services (2014). The Surgeon General’s Call to Action to Prevent Skin Cancer.

[5] Barrett F., Usher K., Woods C., Conway J. (2019). Sun Protective Behaviours during Maximum Exposure to Ultraviolet Radiation When Undertaking Outdoor Activities: An Integrated Literature Review. J. Public Health.

[6] Andersen P.A., Buller D.B., Walkosz B.J., Scott M.D., Beck L., Liu X., Abbott A., Eye R., Cutter G. (2017). A Randomized Trial of an Advanced Sun Safety Intervention for Vacationers at 41 North American Resorts. J. Health Commun..

[7] Glanz K., Geller A.C., Shigaki D., Maddock J.E., Isnec M.R. (2002). A Randomized Trial of Skin Cancer Prevention in Aquatics Settings: The Pool Cool Program. Health Psychol..

[8] National Golf Foundation Golf Industry Facts 2020. https://www.ngf.org/golf-industry-research/.

[9] (2020). U.S. Census Bureau State Population Totals: 2010–2019. https://www.census.gov/data/datasets/time-series/demo/popest/2010s-state-total.html.

[10] del Boz J., Fernández-Morano T., Padilla-España L., Aguilar-Bernier M., Rivas-Ruiz F., de Troya-Martín M. (2015). Skin Cancer Prevention and Detection Campaign at Golf Courses on Spain’s Costa Del Sol. Actas Dermosifiliogr..

[11] Dennis L.K., Vanbeek M.J., Beane Freeman L.E., Smith B.J., Dawson D.V., Coughlin J.A. (2008). Sunburns and Risk of Cutaneous Melanoma: Does Age Matter? A Comprehensive Meta-Analysis. Ann. Epidemiol..

[12] Downs N.J., Schouten P.W., Parisi A.V., Turner J. (2009). Measurements of the Upper Body Ultraviolet Exposure to Golfers: Non-Melanoma Skin Cancer Risk, and the Potential Benefits of Exposure to Sunlight. Photodermatol. Photoimmunol. Photomed..

[13] Hanke C.W., Zollinger T.W., O’Brian J.J., Bianco L. (1985). Skin Cancer in Professional and Amateur Female Golfers. Phys. Sportsmed..

[14] Sung H., Slocum A.C. (2006). UV Radiation Exposure to Body Sites of Golfers and Effects of Clothing. Fam. Consum. Sci. Res. J..

[15] McLeroy K.R., Bibeau D., Steckler A., Glanz K. (1988). An Ecological Perspective on Health Promotion Programs. Health Educ. Q..

[16] Klein K.J., Sorra J.S. (1996). The Challenge of Innovation Implementation. Acad. Manag. Rev..

[17] Courtney J., Handley E., Pagoto S., Russell M., Conroy D.E. (2021). Alcohol Use as a Function of Physical Activity and Golfing Motives in a National Sample of United States Golfers. Nutrients.

[18] Fitzpatrick T.B. (1988). The Validity and Practicality of Sun-Reactive Skin Types I through VI. Arch. Dermatol..

[19] Glanz K., Yaroch A.L., Dancel M., Saraiya M., Crane L.A., Buller D.B., Manne S., O’Riordan D.L., Heckman C.J., Hay J. (2008). Measures of Sun Exposure and Sun Protection Practices for Behavioral and Epidemiologic Research. Arch. Dermatol..

[20] Eason C.D., Rundle C., Dunnick C.A., Hugh J., Dellavalle R.P. (2020). National Trends in Free Public Sunscreen Dispensers. J. Am. Acad. Dermatol..

[21] New Partnership Announced at Outdoor Pools and Public Golf Courses. https://www.siouxfalls.org/news/2019/August/07/partnership.

[22] Officials Unveil Free Sunscreen Dispensers at Local Golf Courses. https://patch.com/new-york/westislip/officials-unveil-free-sunscreen-dispensers-local-golf-courses.

[23] Wood M., Raisanen T., Polcari I. (2017). Observational Study of Free Public Sunscreen Dispenser Use at a Major US Outdoor Event. J. Am. Acad. Dermatol..

[24] American Society for Dermatologic Surgery SPF for All. https://www.asds.net/medical-professionals/public-service-programs/spf-for-all.

[25] Dixon H.G., Hill D.J., Karoly D.J., Jolley D.J., Aden S.M. (2007). Solar UV Forecasts: A Randomized Trial Assessing Their Impact on Adults’ Sun-Protection Behavior. Health Educ. Behav..

[26] Walkosz B.J., Buller D.B., Andersen P.A., Scott M.D., Liu X., Cutter G.R., Dignan M.B. (2015). Translation of a Ski School Sun Safety Program to North American Ski and Snowboard Schools. Health Promot. Pract..

[27] Brinker T.J., Heckl M., Gatzka M., Heppt M.V., Resende Rodrigues H., Schneider S., Sondermann W., de Almeida E Silva C., Kirchberger M.C., Klode J. (2018). A Skin Cancer Prevention Facial-Aging Mobile App for Secondary Schools in Brazil: Appearance-Focused Interventional Study. JMIR Mhealth Uhealth..

[28] Brinker T.J., Faria B.L., de Faria O.M., Klode J., Schadendorf D., Utikal J.S., Mons U., Krieghoff-Henning E., Lisboa O.C., Oliveira A.C.C. (2020). Effect of a Face-Aging Mobile App-Based Intervention on Skin Cancer Protection Behavior in Secondary Schools in Brazil: A Cluster-Randomized Clinical Trial. JAMA Dermatol..

[29] Chambergo-Michilot D., Tellez W.A., Becerra-Chauca N., Zafra-Tanaka J.H., Taype-Rondan A. (2020). Text Message Reminders for Improving Sun Protection Habits: A Systematic Review. PLoS ONE.

[30] Mayer J.A., Lewis E.C., Eckhardt L., Slymen D., Belch G., Elder J., Engelberg M., Eichenfield L., Achter A., Nichols T. (2001). Promoting Sun Safety among Zoo Visitors. Prev. Med..

[31] Crutzen R., Ruiter R. (2015). Interest in Behaviour Change Interventions: A Conceptual Model. Eur. Health Psychol..

[32] Buller D.B., Andersen P.A., Walkosz B.J., Scott M.D., Beck L., Cutter G.R. (2017). Effect of an Intervention on Observed Sun Protection by Vacationers in a Randomized Controlled Trial at North American Resorts. Prev. Med..

[33] Geller A.C., Glanz K., Shigaki D., Isnec M.R., Sun T., Maddock J. (2001). Impact of Skin Cancer Prevention on Outdoor Aquatics Staff: The Pool Cool Program in Hawaii and Massachusetts. Prev. Med..

